# Accumulation of Anthocyanins in Detached Leaves of *Kalanchoë blossfeldiana*: Relevance to the Effect of Methyl Jasmonate on This Process

**DOI:** 10.3390/ijms24010626

**Published:** 2022-12-30

**Authors:** Marian Saniewski, Joanna Szablińska-Piernik, Agnieszka Marasek-Ciołakowska, Joanna Mitrus, Justyna Góraj-Koniarska, Lesław B. Lahuta, Wiesław Wiczkowski, Kensuke Miyamoto, Junichi Ueda, Marcin Horbowicz

**Affiliations:** 1 The National Institute of Horticultural Research, Konstytucji 3 Maja 1/3, 96-100 Skierniewice, Poland; 2 Department of Plant Physiology, Genetics and Biotechnology, University of Warmia and Mazury in Olsztyn, Oczapowskiego 1A, 10-719 Olsztyn, Poland; 3 Institute of Biological Sciences, Siedlce University of Natural Sciences and Humanities, Prusa 14, 08-110 Siedlce, Poland; 4 Department of Chemistry and Biodynamics of Food, Institute of Animal Reproduction and Food Research of the Polish Academy of Sciences, Tuwima 10, 10-748 Olsztyn, Poland; 5 Faculty of Liberal Arts, Sciences and Global Education, Osaka Metropolitan University, 1-1 Gakuen-cho, Naka-ku, Sakai 599-8531, Osaka, Japan; 6 Department of Biological Science, Graduate School of Science, Osaka Prefecture University, 1-1 Gakuen-cho, Naka-ku, Sakai 599-8531, Osaka, Japan

**Keywords:** *Kalanchoë blossfeldiana*, anthocyanins, leaves, methyl jasmonate, polar metabolites, histology

## Abstract

Accumulation of anthocyanins in detached leaves and in excised stems of *Kalanchoë blossfeldiana* kept under natural light conditions in the presence or absence of methyl jasmonate (JA-Me) was investigated. When the abaxial surface of detached leaves was held lower than the adaxial surface (the normal or natural position) under natural light conditions, anthocyanins were not accumulated on the abaxial side of the leaves. In contrast, when the adaxial surface of detached leaves was held lower than the abaxial surface (inverted position), anthocyanins were highly accumulated on the abaxial side of the leaves. These phenomena were independent of the growth stage of *K. blossfeldiana* as well as photoperiod. Application of JA-Me in lanolin paste significantly inhibited anthocyanin accumulation induced on the abaxial side of detached leaves held in an inverted position in a dose-dependent manner. Anthocyanin accumulation in the excised stem in response to natural light was also significantly inhibited by JA-Me in lanolin paste. Possible mechanisms of anthocyanin accumulation on the abaxial side of detached *K. blossfeldiana* leaves held in an inverted position under natural light conditions and the inhibitory effect of JA-Me on this process are described. The accompanying changes in the content of primary metabolites and histological analyses were also described.

## 1. Introduction

*Kalanchoë blossfeldiana* Poelln. is succulent belonging to the short day plant species family [1] (Supplementary Figure S1). A physiological feature of succulents is the opening of stomata and the accumulation of organic acids at night, and the closing of the stomata and the gradual decline of organic acids during the day—referred to as crassulacean acid metabolism (CAM) [2]. Daylight-induced decarboxylation of organic acids controls the size of stomata and causes them to close during the day [3]. Malate and citrate obtained during dark CO_2_ fixation accumulate in the vacuole [4,5,6]. Details of this phenomenon have been recently described [7]. 

Leaves of *K. blossfeldiana* growing under long-day conditions accumulate large amounts of soluble phenolic compounds, unlike those growing under short-day conditions [8]. The process of inflorescence development from flower initiation to anthesis in this plant was described by Hayashi and Konishi [9]. Previously, it was shown that anthocyanin accumulation in the leaves and stems of *K. blossfeldiana* is a consequence of the photoperiodic response [10].

Anthocyanins are glycosidic derivatives of anthocyanidin, and the major anthocyanidins in plants are cyanidin, pelargonidin, peonidin, delphinidin, petunidin, and malvidin [11]. Their structure differs mainly in the number and position of hydroxyl groups, which are glycosylated with glucose, galactose, rhamnose, arabinose, or xylose. Cyanidin glycosides are most common in plants [12], and in *K. blossfeldiana*, cyanidin-3-monoglucoside and cyanidin-3,5-diglucoside have been shown to be the main anthocyanin pigments [13].

Anthocyanins in plants are synthesized through the general flavonoid pathway, is comprised of three molecules of malonyl-CoA and one molecule of 4-coumaroyl-CoA, the latter of which is derived from phenylalanine or tyrosine [14]. Phenylalanine ammonia-lyase (PAL) is the enzyme that catalyzes the elimination of ammonia from l-phenylalanine to give *trans*-cinnamate, which can be a precursor of anthocyanins, flavonols, lignins, proanthocyanidins, and other compounds [12]. Subsequently, dihydrokaempferol produced from naringenin by the action of flavanone 3-hydroxylase is converted to colorless leucoanthocyanidins with dihydroflavonol reductase. Then the conversion of leucoanthocyanidins to anthocyanidins is catalyzed by anthocyanidin synthase. The final step, which determines anthocyanin color, is the formation of anthocyanidin glycosides with the participation of the appropriate glycosyltransferases [12]. 

Foliar anthocyanins are found in epidermal and/or mesophyll cells [15]. Anthocyanins primarily accumulate in photosynthetic cells of tissues exposed to light (epidermis, palisade, and mesophyll cells) [16]. In the stems of various plant species, JA-Me stimulates the accumulation of anthocyanins, indicating their potential involvement in the plant defense system against pathogen attack [17,18,19,20,21]. For instance, it was shown that JA-Me in lanolin paste applied to the middle part of the stem of *K. blossfeldiana* strongly stimulated anthocyanin accumulation in the main and lateral shoots below and above the treated site [10]. However, almost no anthocyanin accumulation was observed in the JA-Me-treated stem after the leaves were removed from the plant. Góraj-Koniarska et al. [20] also demonstrated that JA-Me substantially increased anthocyanin accumulation in the roots of intact *K. blossfeldiana* plants. On the other hand, JA-Me slightly stimulated anthocyanin accumulation in the roots after leaf removal. It seems possible that in *K. blossfeldiana* JA-Me increases anthocyanin biosynthesis directly in shoots and roots or raises levels by transporting them or their precursors from leaves to shoots and roots.

In contrast, in the hypocotyl of common buckwheat (*Fogopyrum esculentum* Moench), where anthocyanins are abundant, JA-Me markedly reduced their content while stimulating the accumulation of proanthocyanidins (PAs) [21,22]. They are present in the fruit, bark, leaves, and seeds of plants [23,24]. The biosynthesis of PAs is affected by biotic and abiotic stresses and plant hormones [25]. UV-B radiation caused a significant accumulation of PAs in aspen leaves [26] and birch leaves [27]. PAs may also be part of antioxidant activity in the overall stress response [28,29] and the plant defense response against biotic agents such as fungi, bacteria, viruses, and herbivores [30].

Earlier studies showed that JA-Me had a strong stimulating effect on anthocyanin accumulation in *K. blossfeldiana* in both stems and roots exposed to light [19,20]. Because of the noted phenomenon of anthocyanin accumulation in detached leaves of *K. blossfeldiana*, we undertook a broader study in this area. One of the objectives in these studies was to determine whether the application of JA-Me would result in a further increase in anthocyanin content.

This paper presents experiments on the effect of storing detached leaves in an inverted position and excised stems of *K. blossfeldiana* under natural light conditions on the accumulation of anthocyanins in them. We also describe possible mechanisms of anthocyanin accumulation in detached leaves and the effect of JA-Me on this process, focusing on changes in basic metabolites and histological analyses.

## 2. Results

### 2.1. Accumulation of Anthocyanins in Detached Leaves of K. blossfeldiana

**Figure 1:**
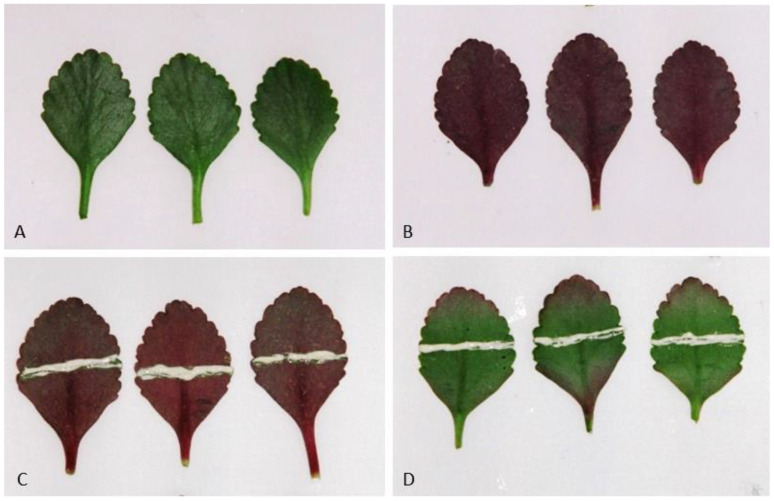
Anthocyanins accumulation in detached leaves of *K. blossfeldiana* when these leaves were kept in the position of the adaxial surface on the lower side (an inverted position) for 4 days in natural light conditions in the presence or absence of methyl jasmonates (JA-Me, 0.5% *w*/*w* in lanolin). The pictures show abaxial surface of detached leaves. (**A**) Initial leaves—detached leaves kept in the position of the adaxial surface underside (inverted position); (**B**) Control—detached leaves kept in the inverted position without treatment; (**C**) Control—detached leaves kept in the inverted position and treated with lanolin only; (**D**) detached leaves kept in the inverted position and treated with 0.5% JA-Me in lanolin.

**Figure 2:**
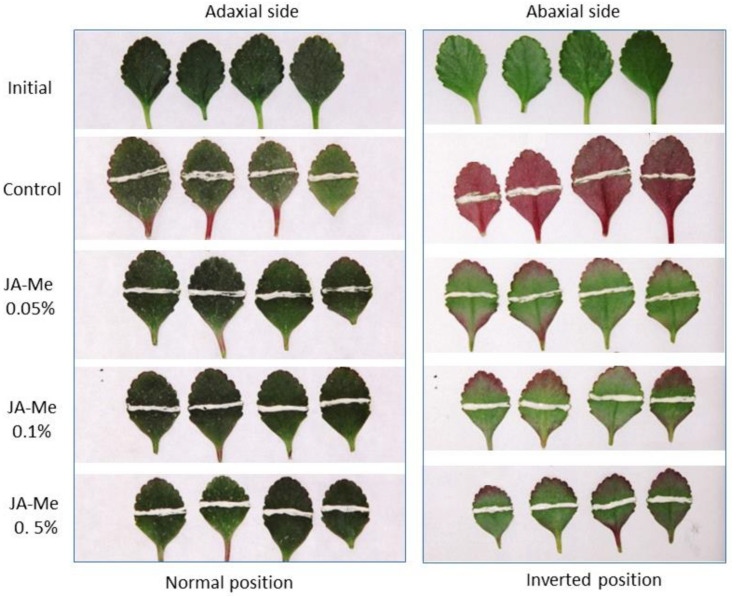
Effect of JA-Me applied to the adaxial or abaxial side of detached leaves of *K. blossfeldiana* on anthocyanin accumulation. After the application of JA-Me to the adaxial side and the abaxial side of detached leaves, the treated leaves were kept in natural light conditions for 4 days in the positions of the abaxial surface on the lower side (normal, natural position) and the adaxial surface on the lower side (inverted position). Pictures contain the abaxial surface (**left**) and the abaxial surface (**right**) of the detached leaves.

**Figure 3:**
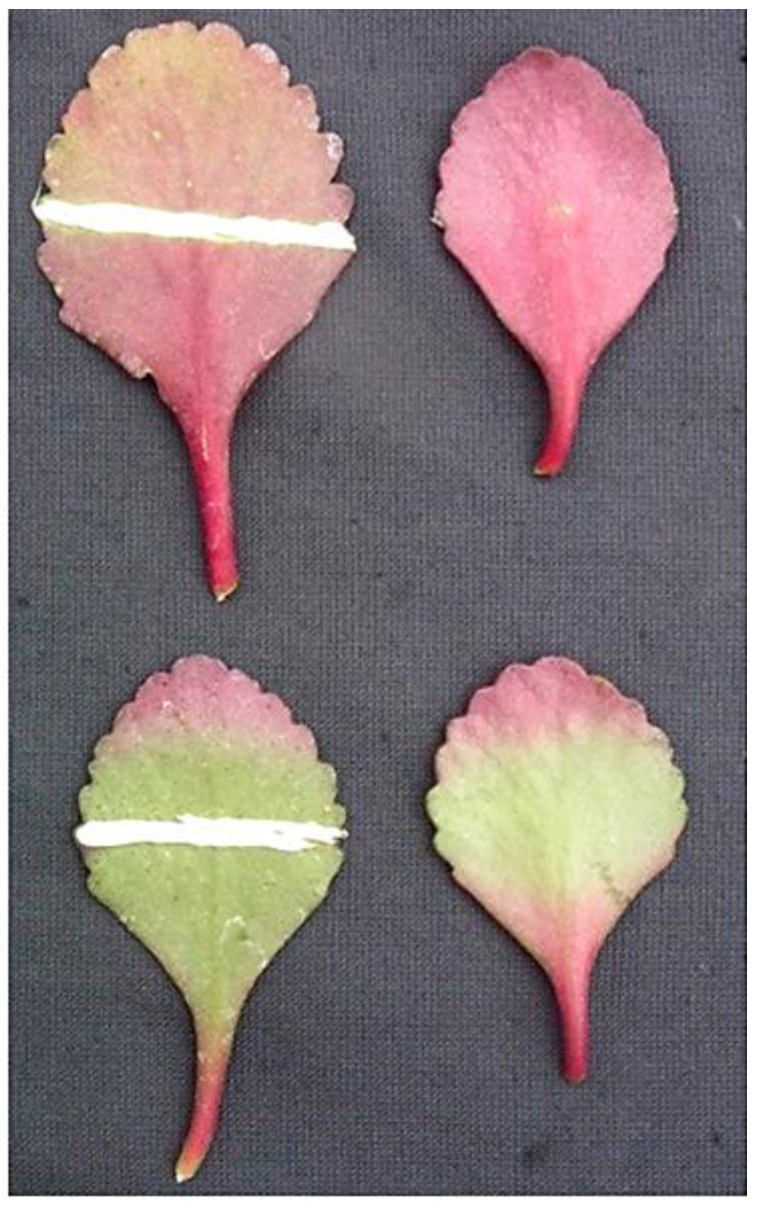
Effect of 0.5% JA-Me in lanolin paste applied on the adaxial or abaxial side of detached leaves of *K. blossfeldiana* on anthocyanins accumulation. The treated leaves were kept in an inverted position (the abaxial side on the lower side) for 4 days in natural light. Upper row: lanolin applied on the abaxial side of detached leaves (**left**) and lanolin applied on the abaxial side (**right**) and kept in an inverted position. Lower row: JA-Me applied on the adaxial side of detached leaves (**left**) and JA-Me applied on the adaxial side (**right**) and kept in an inverted position.

In the experiments described here, it was shown that when the adaxial surface of detached *Kalanchoë blossfeldiana* leaves was held in an inverted position for 4 days, anthocyanins formed on the abaxial side of the leaves. This phenomenon occurred under natural light conditions regardless of the photoperiod of the plant’s growth (Figure 1, Figure 2 and Figure 3). 

This phenomenon did not occur when the abaxial surface of detached leaves was kept on the lower side (a normal or natural position). Application of JA-Me (0.05, 0.1, and 0.5% *w*/*w*) in lanolin paste to the abaxial side of the leaves and then keeping them in an inverted position inhibited anthocyanin accumulation. This occurred below and above the treatment in proportion to the concentrations of JA-Me applied, while application of lanolin alone had no effect on anthocyanin accumulation (Figure 2). Application of JA-Me to the adaxial side of detached leaves held in the normal position (lower side of the abaxial surface) had little effect on the accumulation of anthocyanin. JA-Me applied to the upper (adaxial) side of leaves held in an inverted position also inhibited anthocyanin accumulation on the abaxial side (Figure 3).

**Figure 4:**
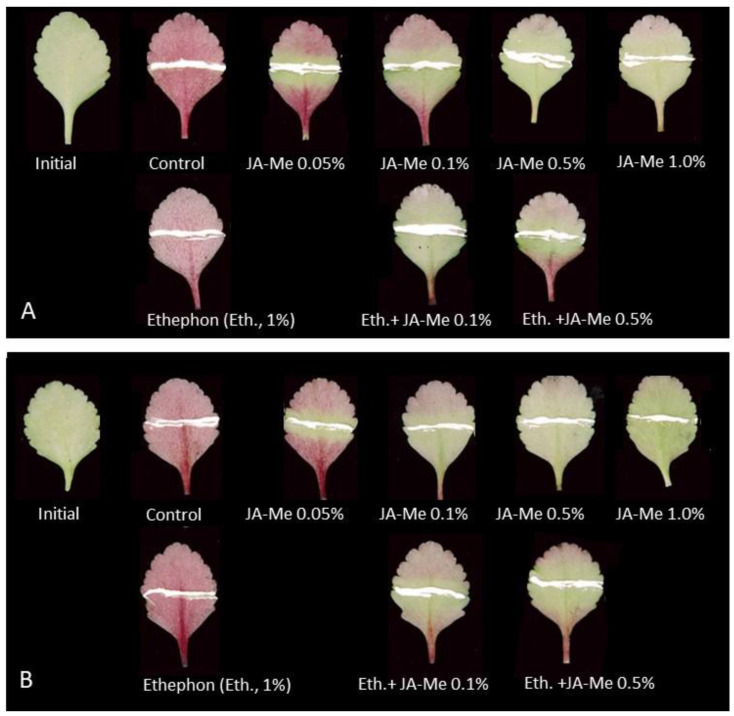
Effect of JA-Me and ethephon applied separately and simultaneously with JA-Me on the abaxial side of detached *K. blossfeldiana* leaves and kept in an inverted position under natural light for 4 days without water (**A**) and on filter paper moistened with water (**B**) on anthocyanin accumulation.

Ethephon applied alone or simultaneously with JA-Me at concentrations of 0.1% and 0.5% did not affect anthocyanin accumulation on the abaxial side of the detached leaves treated and kept in an inverted position compared to the control or JA-Me treatments. These results were similar regardless of whether the leaves were kept in air or on filter paper moistened with water during the experiments (Figure 4).

**Figure 5:**
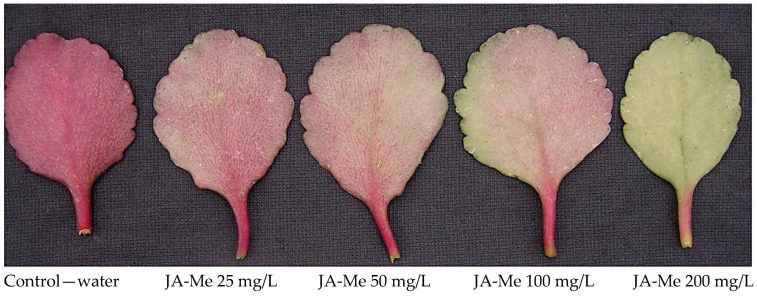
Effect of soaking detached *K. blossfeldiana leaves* in an aqueous solution of JA-Me on anthocyanin accumulation. Various concentrations of JA-Me were used, and the soaking of the leaves lasted for 1 h after which they were kept in an inverted position.

When detached leaves were soaked in JA-Me solution for 1 h and then kept in the inverted position for 4 days in natural light conditions (Figure 5), a significant inhibition of anthocyanin accumulation was observed in a dose-response manner.

**Figure 6:**
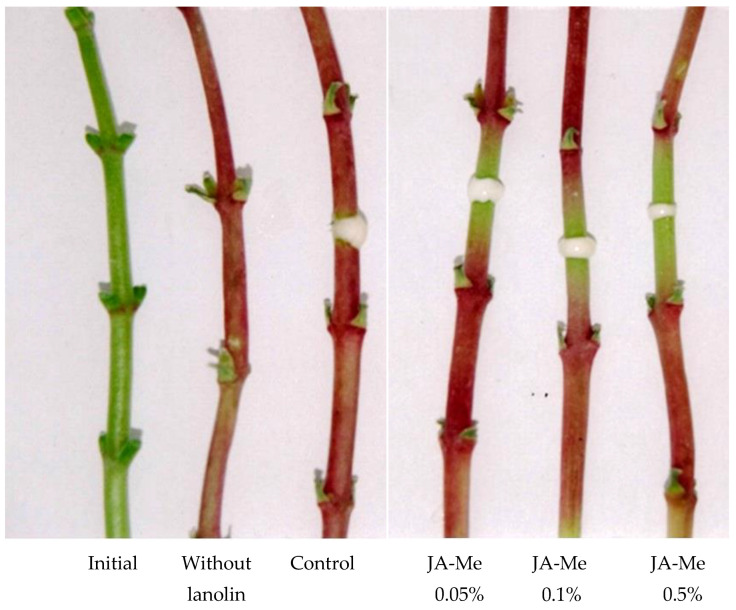
Effect of JA-Me in lanolin on anthocyanin accumulation in excised stems of *K. blossfeldiana*. JA-Me was applied as a lanolin paste, then the treated stem segments were kept in natural light conditions. Pictures were taken 4 days after treatment.

In the detached stems of *K. blossfeldiana,* anthocyanins were formed during a few days of its storage. JA-Me at concentrations of 0.05, 0.1, and 0.5% applied as a ring in the middle of the stem inhibited anthocyanin below and above the treatment proportionally to the concentration applied (Figure 6). This indicates anthocyanin accumulation in stems is as important to JA-Me as that in detached leaves. 

**Figure 7:**
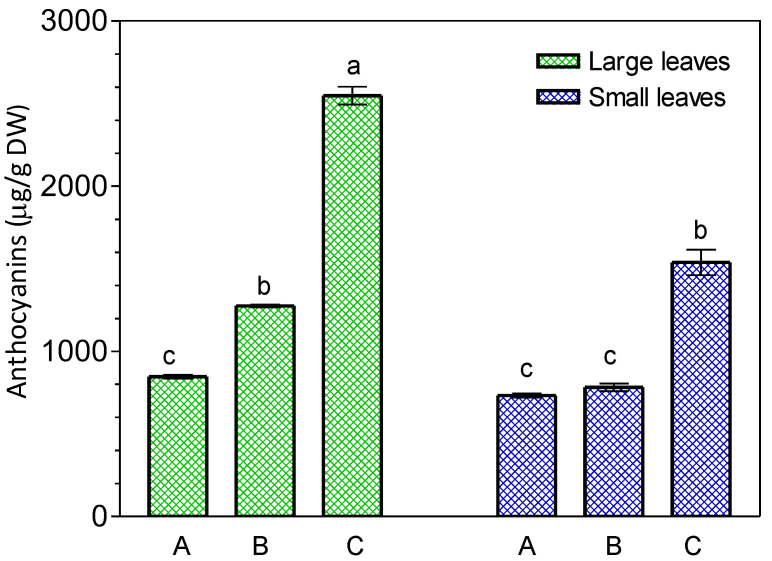
Anthocyanin content in *K. blossfeldiana* leaves on the plant (A) and in the detached leaves kept 4-day in the normal position (B) or kept 4-day in the inverted position (C). Mean results ± standard deviation followed by the same lower letter were not significantly different (*p* < 0.05) according to Tukey’s test.

**Figure 8:**
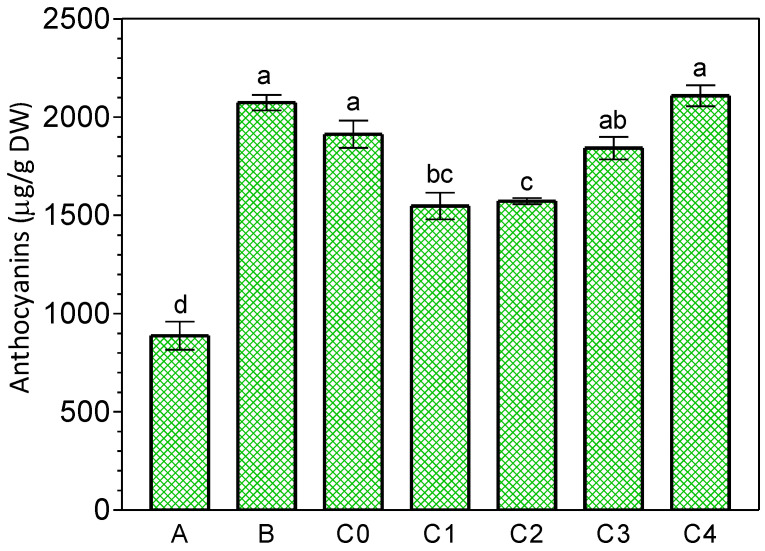
Effect of methyl jasmonate (JA-Me) applied in lanolin paste on the middle part of the excised leaves of *K. blossfeldiana* on total anthocyanin content. A—leaves on the plant; B—leaves kept in the detached position; C0—leaves stored in the inverted position and treated with pure lanolin; C1, C2, C3, and C4—leaves stored in the inverted position and treated with 1% JA-Me, 0.5% JA-Me, 0.25% JA-Me, or 0.1% JA-Me in lanolin paste, respectively. Mean results ± standard deviation followed by the same lower letter were not significantly different (*p* < 0.05) according to Tukey’s test.

**Figure 9:**
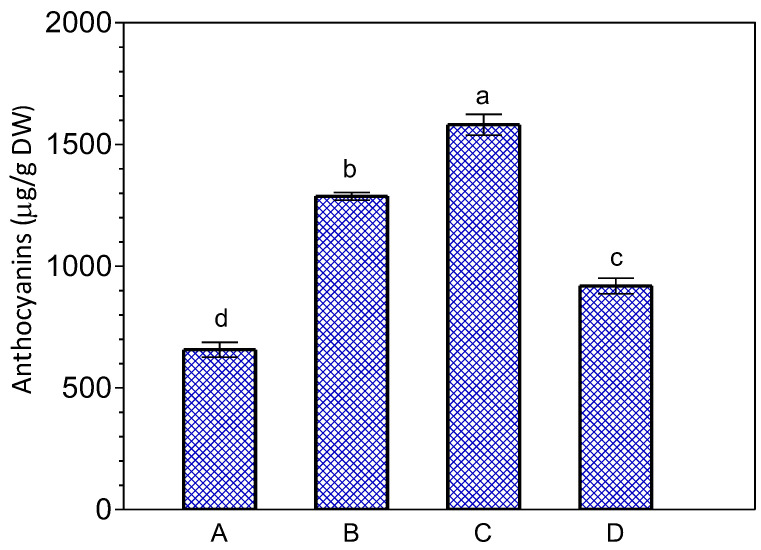
Effect of JA-Me applied in an ethanol-water solution on the anthocyanin content of *K. blossfeldiana* leaves. A—leaves on the plant; B—leaves detached from the plant, treated for 1 h with water, and kept in the inverted position; C—leaves detached from the plant, treated for 1 h with an ethanol-water solution, and kept in the inverted position; D—leaves detached from the plant, treated for 1 h with 10^−3^ M JA-Me in an ethanol-water solution, and kept in the inverted position. Mean results ± standard deviation followed by the same lower letter were not significantly different (*p* < 0.05) according to Tukey’s test.

Older leaves (larger) of *K. blossfeldiana* contained higher levels of anthocyanins compared to younger leaves (smaller) (Figure 7). JA-Me applied in lanolin paste at concentrations of 0.5 and 1.0% inhibited anthocyanin accumulation in the detached leaves as shown in Figure 8. In addition, when the cut leaves were soaked in 10^−3^ M JA-Me in an ethanol-water solution, anthocyanin accumulation was clearly inhibited after being held in the inverted position (Figure 9), whereas JA-Me at a concentration of 10^−4^ M had no such effect. 

Young (smaller leaves) of *K. blossfeldiana* contained higher levels of proanthocyanidins compared to older and larger leaves (Table 1). Increased anthocyanin accumulation in leaves was not accompanied by an increase in proanthocyanidin content compared to green leaves taken directly from the plants (Table 1). In addition, the inhibitory effect of JA-Me on anthocyanin accumulation had no effect on leaf proanthocyanidin content (Table 2).

### 2.2. Primary Metabolites in Detached Leaves of K. blossfeldiana during Accumulation of Anthocyanins

The content of fructose, glucose, sucrose, galactose, and *myo*-inositol was considerably higher in detached leaves of *K. blossfeldiana* kept for 4 days in both the normal and inverted positions under natural light conditions compared to leaves taken directly from the growing plant (Table 3).

The levels of malic, oxalic, lactic, glyceric, and succinic acids were substantially lower in detached leaves kept for 4 days in the light in both the normal and inverted positions compared to leaves on the plant (Table 4). The contents of fumaric and shikimic acids were low and at similar levels in both detached leaves kept in the natural and inverted positions and in leaves on the plant. In contrast, citric acid content was significantly higher in detached leaves than in leaves on the plant (Table 4).

Treatment of detached leaves with JA-Me on their abaxial side and kept in an inverted position for 4 days under light conditions increased the levels of fructose, glucose, and galactose with no effect on sucrose content compared to untreated leaves (Table 5). 

Interestingly, JA-Me significantly increased the glyceric acid content of detached leaves maintained in the inverted position and had little effect on the levels of succinic, fumaric, citric, malic, lactic, and oxalic acids compared to leaves kept in the inverted position (Table 6).

The amino acids valine, alanine, leucine, isoleucine, serine, threonine, and aspartic acid present in *K. blossfeldiana* leaves occurred in very low amounts in both detached leaves and those on the plant. JA-Me had no effect on their content (Supplementary Tables S1 and S2). 

The storage of detached leaves in normal and inverted positions for 4 days caused similar changes in the profiles of polar metabolites in older and younger leaves (Supplementary Figure S3). Principal component analysis (PCA) clearly separated samples of leaves before excision from the plant and after 4 days of storage, according to the PC1 (sharing 87.4% of variability). Moreover, samples of older and younger leaves were also separated according to PC2 (sharing 11.3% of variability). The exposition to the light (natural or inverted) also influenced sample separation (left top and bottom corners on Figure S3A, for younger and older leaves, respectively). The major metabolites, discriminating samples were malate, citrate, sucrose, and glycerate (Figure S3, Table 3).

Treatment of detached leaves with JA-Me on their lower side and kept in an inverted position for 4 days under light conditions affected metabolic profiles (Supplementary Figure S4). Hierarchical cluster analysis (HCA) clearly separated samples of control (before excision from the plant) from detached leaves. Moreover, detached leaves in an inverted position and treated with lanolin paste were grouped in the second cluster, whereas all JA-Me-treated leaves were grouped in the third one. 

### 2.3. Histological Observations of Detached Leaves of K. blossfeldiana during Anthocyanin Accumulation

**Figure 10:**
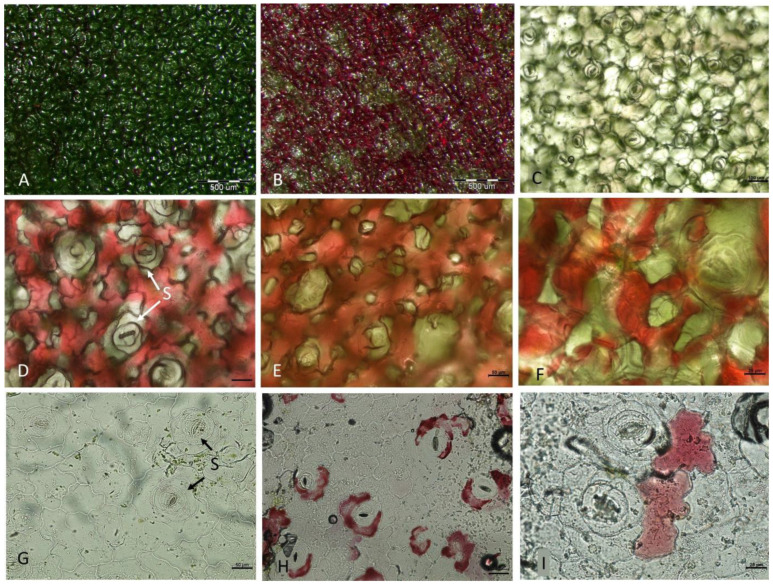
Bright-field microscopy of the leaf surface of *K. blossfeldiana*. (**A**,**B**) Detached leaves were kept for 4 days in an inverted position in natural light conditions. (**A**) Adaxial side of the leaf blade. (**B**) Abaxial side of the leaf blade shows a local occurrence of anthocyanins. (**C**,**G**) Abaxial side of the leaf at day 0 after leaf detachment. (**D**–**F**) Abaxial side of the leaf kept for 4 days in an inverted position in natural light conditions at higher magnification showing local occurrence of anthocyanins. (**D**) Abaxial surface of leaves without additional interference. (**E**,**F**) Abaxial surface of leaves after removal of epidermis. (**G**) Abaxial epidermis with stomata isolated from the control leaf. (**H**,**I**). Abaxial epidermis removed from the leaf was kept for 4 days in an inverted position with local occurrences of anthocyanins. S—stomata. Scar bars: (**A**,**B**) 500 μm, (**C**) 100 μm, (**D**,**E**,**G**) 50 μm, (**F**,**I**) 25 μm, (**H**) 50 μm.

**Figure 11:**
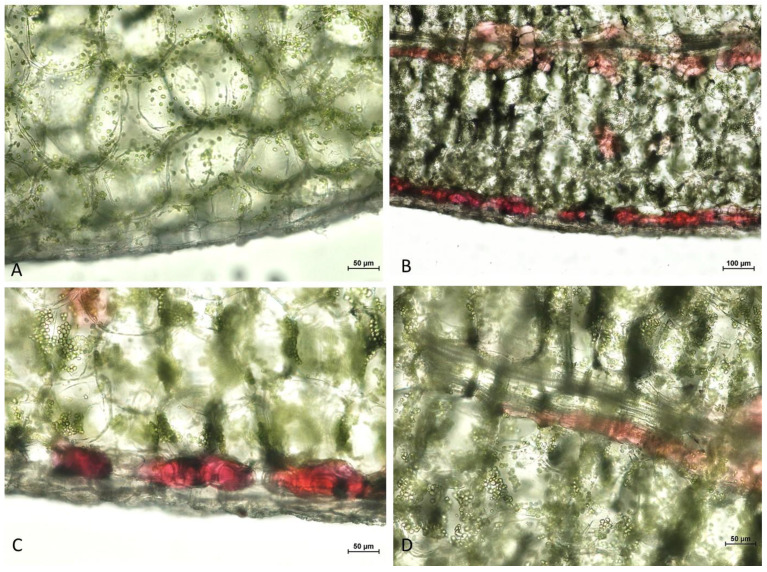
Freehand cross-section of *K. blossfeldiana* leaves indicating local occurrence of anthocyanins. (**A**–**C**) Lower/Abaxial part of the leaf blade. (**A**) control—day 0 after leaf detachment. (**B**–**D**) Detached leaves were kept for 4 days in an inverted position in natural light conditions. (**B**,**C**) Local accumulation of anthocyanins in sub-epidermal mesophyll cells. (**B**,**D**) Occurrence of anthocyanins in mesophyll cells of the vascular bundle.

The color of *K. blossfeldiana* leaves taken directly from the growing plant was the same on both surfaces, devoid of anthocyanins (Supplementary Figure S2). In detached leaves kept upside down against the light, local occurrence of anthocyanin forming a variegation pattern was observed in the abaxial leaf surface, while the color of the adaxial side of the leaf remained unchanged/green (Figure 10). Stomata were present in the adaxial and abaxial epidermis. Anthocyanins were observed in sub-epidermal mesophyll cells in leaves where the epidermis has been removed from their abaxial side (Figure 10E,F). In isolated abaxial epidermis, anthocyanins were found primarily in close proximity to the stomata guard cells (Figure 10H,I). On cross-sections through the lamina, the structure of the mesophyll was homogenous without differentiation into palisade and spongy mesophyll. Anthocyanins were found in sub-epidermal mesophyll cells and mesophyll cells of the vascular bundle, whereas there were no anthocyanins in the upper (adaxial) leaf tissues (Figure 11).

## 3. Discussion

The following question arises: why do anthocyanins form on the underside of detached leaves of *Kalanchoë blossfeldiana,* held upside down against the light? It seems that in *K. blossfeldiana* the epidermis of the lower side of the leaves plays an important role in light perception in terms of the formation and accumulation of anthocyanins there. Schwabe [31] showed that the leaf epidermis in this species plays a significant role in photoperiodic perception, but it is difficult to determine whether the epidermal feature is exerted through stomatal control or through some direct perception by the epidermal cells themselves. Woo-Gyu et al. [32] showed that the density of stomata on the lower surface of *K. blossfeldiana* leaves is higher compared to the upper surface. It is also known that in *K. blossfeldiana,* the upper epidermis is made of polyhedral (hexagonal and pentagonal) cells and contains fewer stomata than the lower epidermis, whose cells have folded walls [33]. Recently, Laskar et al. [34] confirmed that the density of stomata on the lower surface of *K. blossfeldiana* leaves is higher than on the upper surface and showed that the density of epidermal cell arrangement is significantly higher on the lower surface of the leaves. 

It is interesting to note that the response of leaves detached from the plant and kept in a normal (natural) and inverted position for 4 days was similar compared to leaves on the plant. Thus, the accumulation of anthocyanins on the lower side of detached leaves kept in an inverted position was not accompanied by changes in the content of most carbohydrates and acids (except malic acid and citric acid) or other metabolites whose presence was confirmed by GC-MS. In general, the contents of some metabolites in the leaves that were kept for 4 days were higher, some were lower, and some had no effect compared to the leaves on the plant.

In the leaves of the *Kalanchoë* genus, the main acids are malic and citric, as was previously shown [35]. The decrease in malic acid content in detached leaves of *K. blossfeldiana* is mainly due to the fact that the plant belongs to the CAM species. These species attach carbon dioxide to phosphoenolpyruvate at night and then convert it to malic acid, accumulating it in the vacuole. During the day, they use CO_2_ for photosynthesis, which results in an increase in monocarbohydrate content. This metabolic route has been confirmed by the results obtained. It was calculated that there are close correlations between decreases in malic acid content and increases in monosaccharide content and their total contents (Supplementary Table S3). This indicates that basic physiological processes are still taking place in leaves that have been detached from the plant and kept for 4 days. 

The presence of glyceric acid (2,3-dihydroxypropanoic acid) in *K. blossfeldiana* leaves was confirmed for the first time. To date, there is little information on the role of glyceric acid in plants and the course of its biosynthesis, although it has been found in some plants [36,37]. Probably the d-glyceric acid is derived from 3-phospho-d-glyceraldehyde by dehydrogenation to 3-phospho-d-glyceric acid and loss of a phosphate group [38]. Another suggestion is that glyceric acid is produced by the oxidation of glycerol [39]. The decline in its level after a 4-day holding of detached *K. blossfeldiana* leaves was independent of the conditions of this process. These results may indicate that the decrease in glyceric acid content may be related to the stress caused by detaching leaves from the plant. On the other hand, the increase in glyceric acid under JA-Me is hardly explainable. 

It is not clarified why JA-Me stimulates anthocyanin formation in shoots of intact *K. blossfeldiana* plants [10], but it inhibits anthocyanin accumulation in detached leaves held upside down under natural light. It was previously shown that JA-Me induces stomatal closure in *Arabidopsis* guard cells through a stimulatory effect on abscisic acid biosynthesis [40,41,42]. Thus, the involvement of endogenous ABA induced by JA-Me in stomatal closure is most likely related to the inhibition of anthocyanin biosynthesis in detached *K. blossfeldiana* leaves held upside down on the light.

Jasmonates (JAs) have been well known to function as messengers in the biosynthesis of secondary metabolites, including anthocyanins. JA-Me has been shown to stimulate anthocyanin accumulation in wild-type *Arabidopsis thaliana* [43], in the stems and leaves of tulips [18], in cultures of *Vaccinium pahalae* [44], and in other plants. For instance, JA-Me vapor stimulated anthocyanin production in 5-day-old hypocotyls of soybean seedlings growing in the light but inhibited their accumulation in etiolated seedlings [45]. The authors suggested that the reason for this phenomenon may be that two different mechanisms regarding anthocyanin biosynthesis occur in the light and in the dark [45]. Xie et al. [46] documented that anthocyanin accumulation in sprouts of tumorous stem mustard (*Brassica juncea*) can be enhanced by sucrose under light conditions and by JA-Me under light and dark conditions.

Factors resulting from abiotic stresses (wounds, drought, osmotic stress, and others) and biotic stresses also stimulate the biosynthesis of JAs in plants [47]. Thus, the formation of anthocyanins in the epidermal cells and in the layer of mesophyll cells located directly under the epidermis of the detached leaves and detached stem in *K. blossfeldiana* may be a consequence of the increased level of endogenous JAs.

However, it seems that the stimulation of JAs biosynthesis as a result of drought stress is not the cause of anthocyanin accumulation in detached leaves and excised stems of *K. blossfeldiana*. It is known that species belonging to the Crassulaceae (CAM) family are considered insensitive to water deficiency [48]. Thus, the direct factor causing anthocyanin accumulation on the underside of detached leaves held inversely to their natural position and the cut stem stored in the light is unknown. Meanwhile, the application of exogenous JA-Me to detached leaves and stems resulted in an excess of JA in tissues below and above the treatment site, which was probably responsible for the inhibition of anthocyanin accumulation. This is supported by the previously stated evidence that jasmonic acid (JA) and JA-Me can be long-distance transported in the plant [49,50,51].

It should be mentioned that anthocyanins did not accumulate within 4–10 mm of the infected areas in detached leaves of *Crassula multicava* and *K. blossfeldiana* with disease symptoms caused by *Pestalotia* sp. and held in natural light [52,53]. Following the infection of the leaves with the vein, there was a lack of anthocyanin accumulation on the vein, which was clearly visible at some distance from the infected area. It is believed that this phenomenon is caused by the pathogen’s secretion of biologically active compounds, which may be jasmonates. 

Previously, Horbowicz et al. [21,22,54] showed that exogenously applied JA-Me inhibited anthocyanin biosynthesis and accumulation in the hypocotyl of etiolated seedlings of common buckwheat (*Fagopyrum esculentum*). The phenomenon was observed in experiments with various methods of JA-Me treatment. JA-Me also inhibited anthocyanins accumulation in etiolated seedlings of soybean [45]. The stimulating effect of ethephon on anthocyanin accumulation has been reported [55,56]. However, it seems that the application of JA-Me not affect this process in *K. blossfeldiana*. 

In the near future, further intensive research will be necessary to elucidate the mechanisms involved in the accumulation of anthocyanins in the presence or absence of JA-Me, as well as the effect of the method of keeping the leaves after detaching from the plant. These will concern both the composition of anthocyanins present in *K. blossfaldiana* leaves and the sites of their accumulation in the leaf, as well as the activity of enzymes involved in their biosynthesis.

## 4. Materials and Methods

### 4.1. Plant Materials and the Application of Methyl Jasmonate (JA-Me)

Plants of *Kalanchoë blossfeldiana* grown in a greenhouse of the National Institute of Horticultural Research in Skierniewice, Poland (51°57′50.6″ N, 20°10′15.2″ E) were used. The shoot cuttings were taken in March for rooting in a mixture of soil, peat moss, and sand (2:1:1) in a greenhouse under natural conditions, and then replanted separately in pots in the same growing medium. Three- to five-month-old plants of *K. blossfeldiana* having green leaves and stems were used for further experiments with excised leaves (large, older (40 mm width) and small, younger (25 mm width), and an excised stem after removal of leaves and cut at the level of medium (Figure 6; Supplementary Figure S2). All experiments were repeated three to five times, using 20 to 25 leaves per treatment and 20 stems.

Experiment A. Detached leaves of *K. blossfeldiana* were treated with JA-Me at concentrations of 0.1, 0.25, 0.5%, and 1.0% in lanolin paste. JA-Me was applied in the middle part of the leaf blade as a 1–2 mm-wide strip on its upper (adaxial) or lower (abaxial) side. The leaves were kept in a normal (abaxial side underside) or inverted position (adaxial side underside) under natural light conditions in the greenhouse at 20–24 °C under ambient light of 30–50 μmol/m^2^/s PPFD. Control leaves were not treated or treated with lanolin only and kept in the same conditions.

Experiment B. Detached leaves of *K. blossfeldiana* were soaked with water and JA-Me in water solutions at concentrations of 25, 50, 100, and 200 mg/L and in JA-Me at a concentration 10^−3^ M and 10^−4^ M in ethanol-water solutions for 1 h and then were kept in an inverted position in natural light conditions in a greenhouse.

Experiment C. Detached leaves of *K. blossfeldiana* were treated with JA-Me and ethephon dissolved in lanolin paste separately and simultaneously on the abaxial side of the leaves. The leaves were then kept for 4 days in an inverted position under natural light conditions without watering and/or on moistened filter paper.

Experiment D. Excised entire stems of *K blossfeldiana*, after removal of leaves and roots, were treated with JA-Me at a concentration of 0.05, 0.1, and 0.5% (*w*/*w* in lanolin) as a 2 mm-wide ring in the middle of the internode in the middle stem and kept in a natural light conditions in a greenhouse. Anthocyanin accumulation was observed morphologically only.

### 4.2. Analyses of Total Anthocyanins and Proanthocyanidins

Total anthocyanin content was measured as described by Mancinelli [57]. Briefly, freeze-dried and pulverized tissues were extracted with acidified (1% HCl, *w*/*v*) methanol for 24 h at room temperature in darkness with occasional shaking. Then the extracts were carefully decanted, and their absorbance was measured at 530 nm and 657 nm. The formula A_530_–0.25A_657_ was used to compensate for the absorption of chlorophyll degradation. Anthocyanin content was calculated as cyanidin-3-glucoside using 29,600/M x cm as molecular extinction coefficient.

For the determination of total proanthocyanidins, a method based on their hydrolysis to anthocyanidins in HCl-butanol solution was used [58,59]. A freeze-dried and pulverized sample was vortexed with 3.8 mL mixture of n-butanol and concentrated HCl (95:5, *v*/*v*) and 0.2 mL of 2% (*w*/*v*) NH_4_Fe(SO_4_)_2_ · 12 H_2_O in 2 M HCl and centrifuged, and then absorbances were measured at 550 nm. The mixture was then heated in a heating block at 95 °C for 60 min. After cooling and centrifugation, absorbance at 550 nm was measured. The absorbance values of the reaction mixtures before hydrolysis were subtracted from the final absorbance after hydrolysis. Absorbance values were converted to proanthocyanidin equivalents using the molar absorption coefficient of cyanidin chloride in a 5% HCl-butanol solution. 

Reagents including methanol, hydrochloric acid, *n*-butanol, and NH_4_Fe(SO_4_)_2_ · 12 H_2_O were purchased from POCH S.A. (Gliwice, Poland). The absorbance was measured using a Rayleigh type UV-1800 UV/Vis spectrophotometer (Beijing Beifen-Ruili Analytical Instrument Co., Ltd., Nanjing, China)

### 4.3. Polar Metabolite Analyses

Polar metabolites were extracted from freeze-dried and pulverized leaf tissues according to the method described earlier [60]. Briefly, the polar metabolites were extracted from samples with a mixture of methanol:water (1:1, *v*/*v*, containing ribitol as an internal standard) at 70 °C for 30 min. Homogenates were centrifuged, and aliquots of the clear supernatant were extracted with chloroform to remove non-polar compounds. The polar fraction was concentrated to dryness in a speed vacuum rotary evaporator (JWElectronic, Warsaw, Poland). The metabolites were derivatized with *O*-methoxamine hydrochloride and then with *N*-methyl-*N*-trimethylsilyl-trifluoroacetamide. The resulting trimethylsilyl derivatives were analyzed on a ZEBRON ZB-5MSi Guardian capillary column (Phenomenex, Torrance, CA, USA).

Metabolites were identified by comparison of their retention time, retention indices, and mass spectra of original standards purchased from Sigma-Aldrich (Saint Louis, MO, USA) and from the NIST 05 library (National Institute of Standards and Technology, NIST, Gaithersburg, MD, USA). Two gas chromatographs were applied: the GC-2030 Nexis (Shimadzu, Kyoto, Japan) equipped with a flame ionization detector and the GC-2010 coupled with a quadrupole mass spectrometry analyzer (GCMS-QP2010 Plus, Shimadzu, Kyoto, Japan). The details of chromatographic separations were described previously [61]. For the quantitative analyses of identified polar metabolites, the data obtained for the peak areas from the GC-FID analyses were used.

### 4.4. Histological Analyses of the Local Occurrence of Anthocyanins in Leaves

Detached leaves were taken for histological observation at the beginning of the experiment (0 day after leaf detachment, control) and after 4 days of keeping them in an inverted position under natural light conditions. Cross-sections of detached leaves of *K. blossfeldiana* were prepared free-hand using a razor blade. Microscopic preparations were also made of the isolated adaxial and abaxial epidermis, which were observed unstained in a light microscope (Eclipse 80i, Nikon, Tokyo, Japan).

### 4.5. Statitistics 

Analyses were performed in three replicates. An analysis of variance (one-way ANOVA) and Tukey’s post hoc test were used to check the significance of the differences. These calculations and the Pearson’s correlation coefficients were performed using Statistica 12PL software (StatSoft, Tulsa, OK, USA). The results are shown as means ± standard deviation. 

Normalized data were considered using a multivariate statistical analysis (principal component analysis, PCA, and hierarchical cluster analysis, HCA), which was performed in three replicates using COVAIN, a MATLAB toolbox including a graphical user interface (MATLAB version 2013a; Math Works, Natick, MA, USA) [61].

## 5. Conclusions

Detached leaves of *Kalanchoë blossfeldiana* at two stages of development, stored for 4 days in an inverted position under ambient light conditions, accumulated anthocyanins on their abaxial side. Anthocyanins did not accumulate on the adaxial and abaxial sides of detached leaves when they were kept in the normal (natural) position, suggesting that the process of accumulation on the abaxial side of leaves kept in the inverted position is independent of the photoperiod. On the other hand, methyl jasmonate (JA-Me) significantly inhibited anthocyanin accumulation in detached leaves on the abaxial side held in the inverted position. In the present study, we discuss the possible mechanisms involved in anthocyanin accumulation on the abaxial side of detached *K. blossfeldiana* leaves held in an inverted position and the inhibitory effect of JA-Me on this process. 

## Figures and Tables

**Table 1:** Proanthocyanidins content (μM/g DW) in *K. blossfeldiana* leaves on the plant and in leaves detached and kept 4 days in the natural position or kept 4 days in the inverted position. Mean results in columns ± standard deviation (SD) followed by the same letter were not significantly different (*p* < 0.05) according to Tukey’s test.

Leaf Type	Small Leaves	Large Leaves
On plant	13.52 ± 0.38 ^a^	11.56 ± 0.18 ^b^
Kept in the normal (natural) position	13.95 ± 0.33 ^a^	11.81 ± 0.46 ^b^
Kept in the inverted position	14.19 ± 0.09 ^a^	11.12 ± 0.15 ^b^

**Table 2:** Proanthocyanidins content (μMol/g DW) in the leaves of *K. blossfeldiana* on the plant and in leaves detached from the plant and kept for 4 days in the normal (natural) position, kept for 4 days in the inverted position, and kept for 4 days in the inverted position after treatment with various concentrations of methyl jasmonate (JA-Me) in lanolin paste. Mean results ± standard deviation (SD) followed by the same letter were not significantly different (*p* < 0.05) according to Tukey’s test.

Leaf Type	Mean ± SD
On plant	8.39 ± 0.09 ^b^
Kept in the normal (natural) position	7.35 ± 0.27 ^d^
Kept in the inverted position	7.81 ± 0.33 ^cd^
Treated with lanolin and kept in the inverted position	9.88 ± 0.17 ^a^
Treated with 1% JA-Me and kept in the inverted position	9.55 ± 0.29 ^a^
Treated with 0.5% JA-Me and kept in the inverted position	9.26 ± 0.19 ^a^
Treated with 0.25% JA-Me and kept in the inverted position	8.20 ± 0.41 ^abcd^
Treated with 0.1% JA-Me and kept in the inverted position	8.42 ± 0.05 ^bc^

**Table 3:** Carbohydrate content (mg/g DW) in *K. blossfeldiana* leaves on the plant and in leaves detached from the plant and kept for 4 days in the normal (natural) position or kept for 4 days in the inverted position. Mean results in rows ± standard deviation followed by the same letter were not significantly different (*p* < 0.05) according to Tukey’s test.

Carbohydrate	Left on Plant	Normal Position	Inverted Position	Left on Plant	Normal Position	Inverted Position
Large leaves				Small leaves		
Fructose	0.95 ± 0.05 ^b^	2.02 ± 0.09 ^a^	2.34 ± 0.15 ^a^	0.72 ± 0.01 ^c^	2.06 ± 0.13 ^a^	2.54 ± 0.15 ^a^
Glucose	0.97 ± 0.05 ^b^	1.88 ± 0.16 ^a^	2.17 ± 0.12 ^a^	0.98 ± 0.04 ^b^	2.64 ± 0.17 ^a^	2.68 ± 0.15 ^a^
Galactose	0.90 ± 0.02 ^b^	1.32 ± 0.08 ^a^	1.55 ± 0.06 ^a^	0.68 ± 0.03 ^c^	1.41 ± 0.13 ^a^	1.59 ± 0.08 ^a^
*myo*-Inositol	0.29 ± 0.01 ^b^	0.61 ± 0.02 ^a^	0.62 ± 0.03 ^a^	0.26 ± 0.01 ^b^	0.67 ± 0.01 ^a^	0.67 ± 0.01 ^a^
Sucrose	2.82 ± 0.05 ^c^	4.00 ± 0.09 ^a^	3.36 ± 0.07 ^b^	1.38 ± 0.12 ^d^	0.73 ± 0.06 ^f^	0.95 ± 0.04 ^e^

**Table 4:** Acid content (mg/g DW) of *K. blossfeldiana* leaves on the plant, and in leaves detached from the plant and kept for 4 days in the normal (natural) position or kept for 4 days in the inverted position. Mean results in rows ± standard deviation followed by the same letter were not significantly different (*p* < 0.05) according to Tukey’s test.

Acid	Left on Plant	Normal Position	Inverted Position	Left on Plant	Normal Position	Inverted Position
Large leaves				Small leaves		
Succinic	0.26 ± 0.01 ^a^	0.14 ± 0.01 ^b^	0.15 ± 0.02 ^b^	0.28 ± 0.01 ^a^	0.11 ± 0.01 ^b^	0.12 ± 0.01 ^b^
Fumaric	0.13 ± 0.02 ^a^	0.09 ± 0.01 ^a^	0.10 ± 0.01 ^a^	0.10 ± 0.01 ^a^	0.08 ± 0.02 ^a^	0.08 ± 0.01 ^a^
Citric	12.99 ± 0.26 ^b^	16.25 ± 0.44 ^a^	17.29 ± 0.57 ^a^	6.59 ± 0.17 ^c^	12.65 ± 0.81 ^b^	14.02 ± 0.56 ^b^
Malic	43.69 ± 4.06 ^ab^	19.22 ± 0.57 ^c^	21.55 ± 1.13 ^c^	32.60 ± 0.30 ^b^	20.72 ± 0.20 ^c^	21.96 ± 0.89 ^c^
Shikimic	0.15 ± 0.02 ^b^	0.10 ± 0.01 ^b^	0.10 ± 0.01 ^b^	0.35 ± 0.04 ^a^	0.27 ± 0.01 ^a^	0.24 ± 0.01 ^a^
Glyceric	0.72 ± 0.06 ^b^	0.05 ± 0.01 ^d^	0.05 ± 0.01 ^d^	2.29 ± 0.01 ^a^	0.20 ± 0.01 ^c^	0.21 ± 0.01 ^c^
Lactic	0.19 ± 0.02 ^a^	0.25 ± 0.08 ^a^	0.19 ± 0.06 ^a^	0.25 ± 0.10 ^a^	0.11 ± 0.07 ^a^	0.18 ± 0.07 ^a^
Oxalic	0.76 ± 0.12 ^a^	0.36 ± 0.09 ^ab^	0.33 ± 0.01 ^b^	0.55 ± 0.02 ^a^	0.32 ± 0.01 ^b^	0.31 ± 0.03 ^b^
Phosphoric	2.94 ± 0.35 ^a^	2.46 ± 0.21 ^a^	2.74 ± 0.16 ^a^	1.81 ± 0.08 ^b^	2.71 ± 0.09 ^a^	3.01 ± 0.08 ^a^

**Table 5:** Effect of methyl jasmonate (JA-Me) applied in lanolin paste on the middle part of the detached leaves of *K. blossfeldiana* on carbohydrate content. A—leaves on the plant; B—leaves detached from plant and kept in the inverted position for 4 days; C0—leaves kept in the inverted position and treated with pure lanolin; C1, C2, C3, and C4—leaves kept in the inverted position and treated with 1% JA-Me, 0.5% JA-Me, 0.25% JA-Me, or 0.1% JA-Me in lanolin paste, respectively. Mean results in rows ± standard deviation followed by the same letter were not significantly different (*p* < 0.05) according to Tukey’s test.

Carbohydrate	A	B	C0	C1	C2	C3	C4
Fructose	1.20 ± 0.02 ^d^	1.88 ± 0.03 ^c^	3.31 ± 0.32 ^c^	12.68 ± 2.43 ^b^	21.74 ± 0.92 ^a^	24.10 ± 1.12 ^a^	13.16 ± 3.49 ^ab^
Glucose	1.35 ± 0.28 ^c^	2.05 ± 0.05 ^c^	3.41 ± 0.26 ^c^	9.56 ± 1.05 ^b^	15.10 ± 0.53 ^a^	16.26 ± 0.82 ^a^	9.19 ± 1.63 ^bc^
Galactose	0.57 ± 0.02 ^c^	1.52 ± 0.03 ^b^	0.52 ± 0.15 ^c^	2.43 ± 0.48 ^ab^	0.55 ± 0.23 ^c^	0.38 ± 0.15 ^bc^	0.97 ± 0.48 ^abc^
Inositol	0.21 ± 0.02 ^a^	0.26 ± 0.01 ^a^	0.26 ± 0.01 ^a^	0.21 ± 0.02 ^a^	0.14 ± 0.01 ^b^	0.15 ± 0.02 ^a^	0.13 ± 0.03 ^a^
Sucrose	3.76 ± 0.10 ^c^	3.66 ± 0.02 ^c^	4.27 ± 0.21 ^b^	6.27 ± 0.46 ^a^	4.45 ± 0.13 ^b^	3.34 ± 0.25 ^bc^	3.84 ± 0.27 ^b^

**Table 6:** Effect of methyl jasmonate (JA-Me) applied in lanolin paste on the middle part of the detached leaves of *K. blossfeldiana* on acid content. A—leaves on the plant; B—leaves detached from plant and kept in the inverted position for 4 days; D0—leaves kept in the inverted position and treated with pure lanolin; D1, D2, D3, and D4—leaves kept in the inverted position and treated with 1% JA-Me, 0.5% JA-Me, 0.25% JA-Me, or 0.1% JAMe in lanolin paste, respectively. Mean results in rows ± standard deviation followed by the same letter were not significantly different (*p* < 0.05) according to Tukey’s test.

Acid	A	B	C0	C1	C2	C3	C4
Succinic	0.21 ± 0.03 ^b^	0.27 ± 0.02 ^b^	0.15 ± 0.01 ^b^	0.35 ± 0.01 ^a^	0.35 ± 0.00 ^ab^	0.37 ± 0.01 ^a^	0.33 ± 0.02 ^ab^
Fumaric	0.13 ± 0.09 ^a^	0.05 ± 0.01 ^a^	0.05 ± 0.01 ^a^	0.08 ± 0.02 ^a^	0.09 ± 0.01 ^a^	0.10 ± 0.02 ^a^	0.09 ± 0.02 ^a^
Citric	9.24 ± 0.17 ^b^	13.27 ± 0.68 ^a^	13.91 ± 0.21 ^a^	8.86 ± 0.68 ^b^	7.80 ± 0.15 ^b^	9.30 ± 0.44 ^b^	9.87 ± 1.04 ^b^
Malic	27.98 ± 0.62 ^a^	13.53 ± 2.33 ^bc^	13.39 ± 5.01 ^abc^	18.81 ± 0.17 ^b^	17.95 ± 0.79 ^b^	9.33 ± 2.94 ^c^	5.78 ± 1.70 ^c^
Shikimic	0.05 ± 0.02 ^a^	0.05 ± 0.01 ^a^	0.05 ± 0.01 ^a^	0.04 ± 0.01 ^a^	0.03 ± 0.01 ^a^	0.03 ± 0.01 ^a^	0.05 ± 0.02 ^a^
Glyceric	0.74 ± 0.02 ^a^	0.05 ± 0.01 ^c^	0.06 ± 0.01 ^c^	0.43 ± 0.08 ^b^	0.48 ± 0.02 ^b^	0.55 ± 0.01 ^b^	0.44 ± 0.06 ^b^
Lactic	0.66 ± 0.10 ^ab^	0.14 ± 0.06 ^cd^	0.11 ± 0.03 ^d^	0.07 ± 0.02 ^d^	0.14 ± 0.02 ^b^	0.13 ± 0.05 ^cd^	0.15 ± 0.03 ^d^
Oxalic	0.55 ± 0.02 ^a^	0.22 ± 0.04 ^b^	0.13 ± 0.02 ^b^	0.13 ± 0.03 ^b^	0.06 ± 0.00 ^b^	0.05 ± 0.03 ^b^	0.06 ± 0.04 ^b^
Phosphoric	1.30 ± 0.08 ^b^	1.42 ± 0.03 ^b^	1.77 ± 0.02 ^a^	0.68 ± 0.16 ^c^	1.03 ± 0.02 ^bc^	1.40 ± 0.05 ^b^	1.93 ± 0.36 ^ab^

## Data Availability

The data presented in this study are available in this article.

## Supplementary Materials

The following supporting information can be downloaded at: https://www.mdpi.com/article/10.3390/ijms24010626/s1.
