# Supplementary material for: Accumulation of Anthocyanins in Detached Leaves of Kalanchoë blossfeldiana: Relevance to the Effect of Methyl Jasmonate on This Process
Source: Int J Mol Sci. 2022 Dec 30;24(1):626. doi: 10.3390/ijms24010626 (PMC9820172; doi:10.3390/ijms24010626)
Supplement: Supplementary file 1 [file ijms-24-00626-s001.zip › ijms-2125117-supplementary.pdf]

Supplementary materials

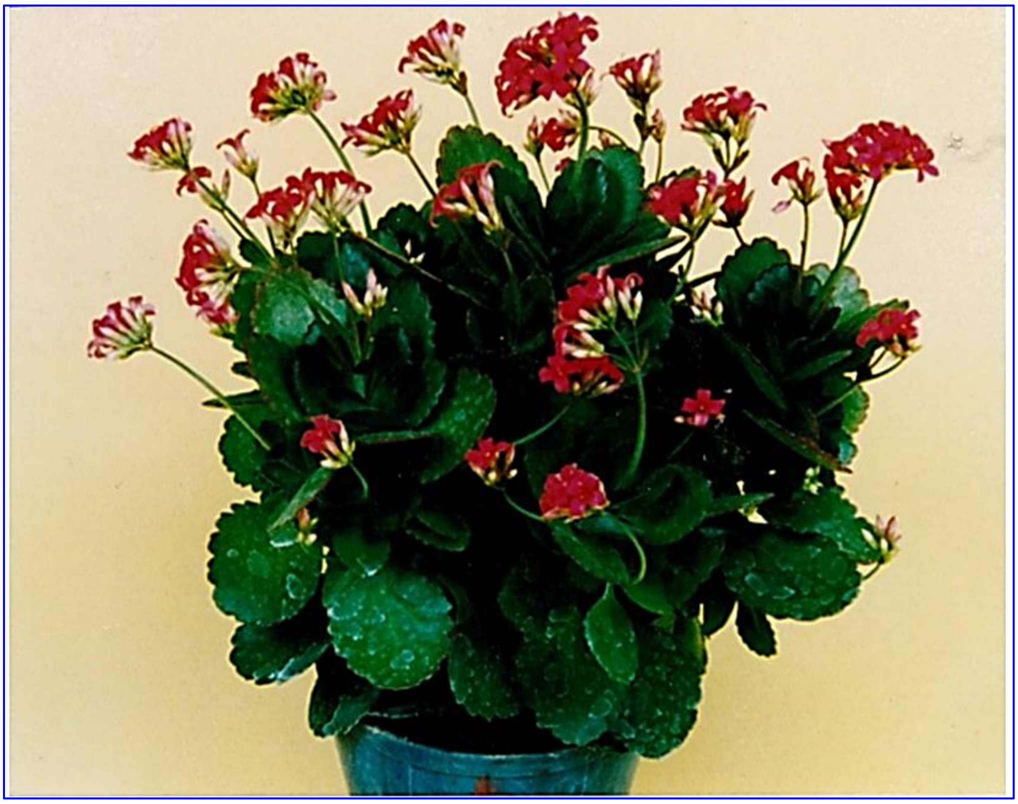

Figure S1. Flowering *Kalanchoë blossfeldiana* plant

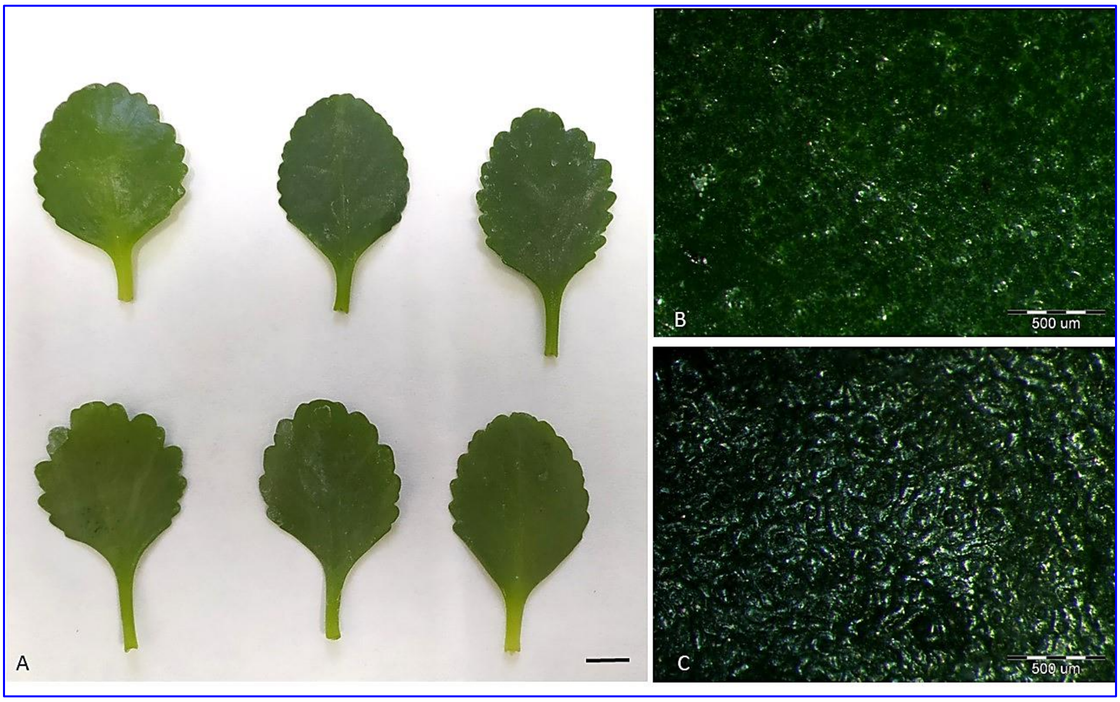

Figure S2. Leaves detached from *K. blossfeldiana* plant at the beginning of the experiment. (A) Top row – adaxial surface of the leaves. Bottom row – abaxial surface of the leaves. Bar represent 10 mm. (B) Adaxial side of the leaf. (C). Abaxial side of the leaf.

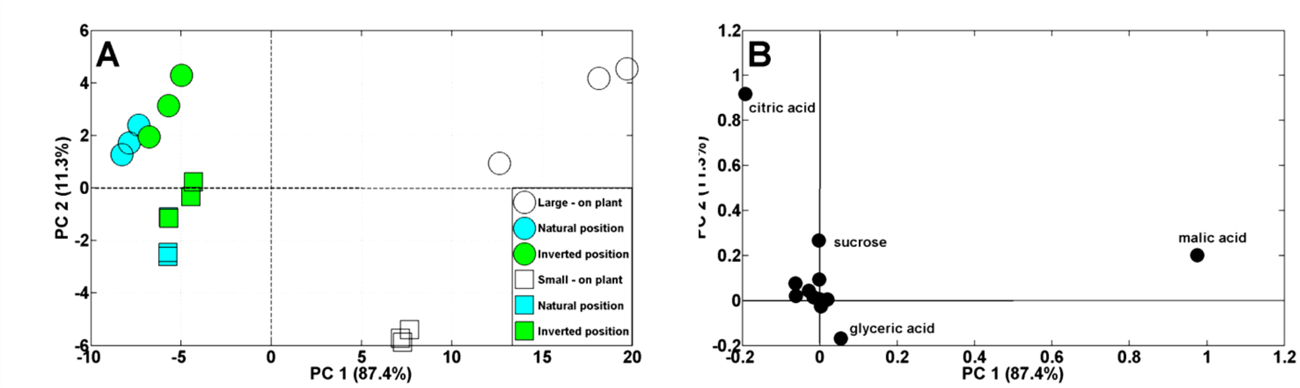

Figure S3. PCA of polar metabolite profiles in small/younger (circles) and large/older (squares) leaves before (empty symbols) and after 4 days of holding under natural light conditions in normal (blue symbols) and inverted (green symbols) position against the light (A). The major discriminating metabolites are shown on B.

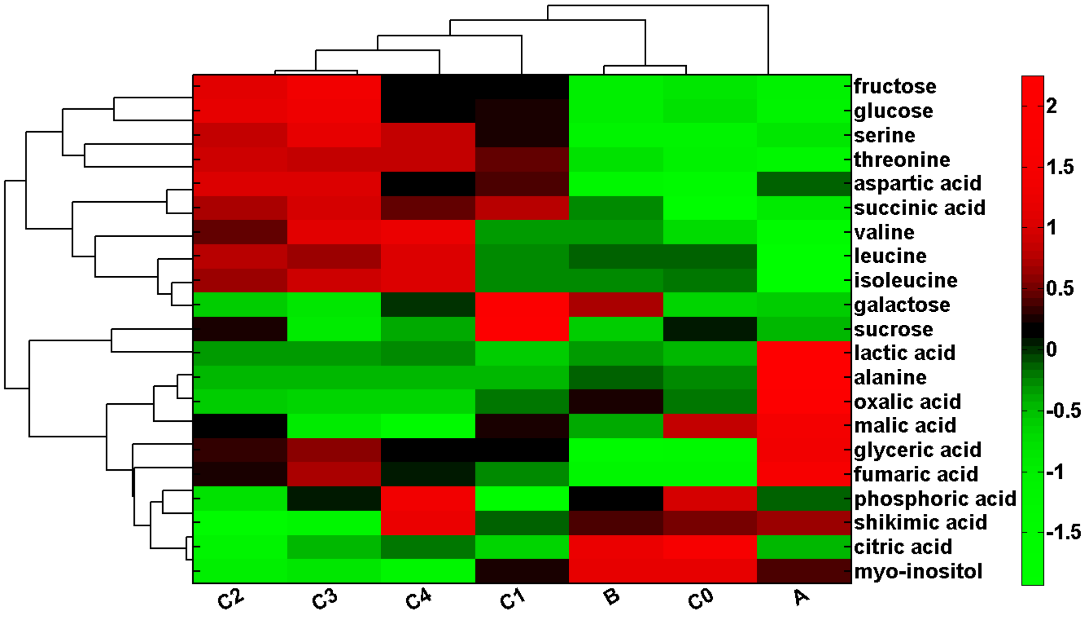

Figure S4. HCA of leaves samples *K. blossfeldiana* on plant (A), and after 4 days of storage of detached leaves kept in an inverted position against the light (B) and treated with lanolin paste containing JA-Me at concentrations of 0, 1.0, 0.5, 0.25 and 0.1% (C0, C1, C2, C3 and C4, respectively)

Table S1. Amino acid content (mg/g DW) in *K. blossfeldiana* leaves on the plant, and in leaves detached and kept for 4 days in the natural position or kept for 4 days in the inverted position. Mean results in rows ± standard deviation followed by the same letter were not significantly different ($p < 0.05$) according to Tukey's test.

Amino acid	On plant	Normal position	Inverted position	On plant	Normal position	Inverted position
	Large leaves			Small leaves		
Valine	0.02 ± 0.01^{a}	0.05 ± 0.01^{a}	0.07 ± 0.02^{a}	0.01 ± 0.01^{a}	0.02 ± 0.01^{a}	0.03 ± 0.01^{a}
Alanine	0.03 ± 0.03^{a}	Nd	Nd	0.06 ± 0.01^{a}	0.02 ± 0.01^{a}	0.03 ± 0.01^{a}
Leucine	Nd	0.09 ± 0.01^{a}	0.08 ± 0.01^{a}	Nd	0.02 ± 0.01^{b}	0.02 ± 0.01^{b}
Isoleucine	0.01 ± 0.01^{b}	0.10 ± 0.02^{a}	0.11 ± 0.02^{a}	0.01 ± 0.01^{b}	0.05 ± 0.02^{ab}	0.06 ± 0.01^{ab}
Serine	0.02 ± 0.01^{a}	0.02 ± 0.01^{a}	0.02 ± 0.01^{a}	0.01 ± 0.01^{a}	0.02 ± 0.01^{a}	0.02 ± 0.01^{a}

Table S2. Effect of methyl jasmonate (JA-Me) applied in lanolin paste on the middle part of the detached leaves of *K. blossfeldiana* on amino acids content. A - leaves on the plant; B -leaves kept in the inverted position for 4 days; C0 - leaves kept in the inverted position and treated with pure lanolin; C1, C2,C3, C4 - leaves stored in the inverted position and treated with 1% JA-Me, 0.5% JA-Me, 0.25% JA-Me or 0.1% JA-Me in lanolin paste, respectively. Mean results in rows ± standard deviation followed by the same letter were not significantly different ($p < 0.05$) according to Tukey's test.

Amino acid	A	B	C0	C1	C2	C3	C4
Valine	0.01 ± 0.01^{a}	0.03 ± 0.01^{a}	0.02 ± 0.01^{a}	0.03 ± 0.01^{a}	0.07 ± 0.02^{a}	0.04 ± 0.01^{a}	0.05 ± 0.01^{a}
Alanine	0.06 ± 0.01^{a}	0.06 ± 0.01^{a}	0.06 ± 0.01^{a}	0.06 ± 0.01^{a}	0.06 ± 0.01^{a}	0.06 ± 0.01^{a}	0.06 ± 0.01^{a}
Leucine	Nd	0.02 ± 0.01^{a}	0.02 ± 0.01^{a}	0.02 ± 0.01^{a}	0.06 ± 0.02^{a}	0.03 ± 0.01^{a}	0.03 ± 0.01^{a}
Isoleucine	Nd	0.04 ± 0.01^{a}	0.04 ± 0.01^{a}	0.04 ± 0.01^{a}	0.11 ± 0.04^{a}	0.06 ± 0.01^{a}	0.07 ± 0.01^{a}
Serine	0.02 ± 0.01^{a}	0.01 ± 0.01^{a}	0.01 ± 0.01^{a}	0.04 ± 0.01^{a}	0.04 ± 0.01^{a}	0.07 ± 0.01^{a}	0.06 ± 0.01^{a}
Threonine	0.02 ± 0.01^{a}	0.03 ± 0.01^{a}	0.03 ± 0.01^{a}	0.07 ± 0.01^{a}	0.08 ± 0.02^{a}	0.08 ± 0.02^{a}	0.08 ± 0.02^{a}
Aspartic acid	0.03 ± 0.01^{a}	0.01 ± 0.01^{a}	0.01 ± 0.01^{a}	0.04 ± 0.01^{a}	0.02 ± 0.01^{a}	0.05 ± 0.01^{a}	0.03 ±0.01^{a}

Table S3. Spearman's correlation coefficients between malic acid and monosaccharide content and their total content in leaves of *K. blossfeldiana* on the plant and detached and kept for 4 days in the light in the inverted and normal (natural) position

	Pearson's correlation coefficient	significance level
Fructose vs. malic acid	-0.809	<0.001
Glucose vs. malic acid	-0.807	<0.001
Galactose vs. malic acid	-0.744	0.001
Total monosaccharides vs. malic acid	-0.818	<0.001
